# Clinical Context–Aware Biomedical Text Summarization Using Deep Neural Network: Model Development and Validation

**DOI:** 10.2196/19810

**Published:** 2020-10-23

**Authors:** Muhammad Afzal, Fakhare Alam, Khalid Mahmood Malik, Ghaus M Malik

**Affiliations:** 1 Department of Software Sejong University Seoul Republic of Korea; 2 Department of Computer Science & Engineering School of Engineering and Computer Science Oakland University Rochester, MI United States; 3 Department of Neurosurgery Henry Ford Hospital Detroit, MI United States

**Keywords:** biomedical informatics, automatic text summarization, deep neural network, word embedding, semantic similarity, brain aneurysm

## Abstract

**Background:**

Automatic text summarization (ATS) enables users to retrieve meaningful evidence from big data of biomedical repositories to make complex clinical decisions. Deep neural and recurrent networks outperform traditional machine-learning techniques in areas of natural language processing and computer vision; however, they are yet to be explored in the ATS domain, particularly for medical text summarization.

**Objective:**

Traditional approaches in ATS for biomedical text suffer from fundamental issues such as an inability to capture clinical context, quality of evidence, and purpose-driven selection of passages for the summary. We aimed to circumvent these limitations through achieving precise, succinct, and coherent information extraction from credible published biomedical resources, and to construct a simplified summary containing the most informative content that can offer a review particular to clinical needs.

**Methods:**

In our proposed approach, we introduce a novel framework, termed Biomed-Summarizer, that provides quality-aware Patient/Problem, Intervention, Comparison, and Outcome (PICO)-based intelligent and context-enabled summarization of biomedical text. Biomed-Summarizer integrates the prognosis quality recognition model with a clinical context–aware model to locate text sequences in the body of a biomedical article for use in the final summary. First, we developed a deep neural network binary classifier for quality recognition to acquire scientifically sound studies and filter out others. Second, we developed a bidirectional long-short term memory recurrent neural network as a clinical context–aware classifier, which was trained on semantically enriched features generated using a word-embedding tokenizer for identification of meaningful sentences representing PICO text sequences. Third, we calculated the similarity between query and PICO text sequences using Jaccard similarity with semantic enrichments, where the semantic enrichments are obtained using medical ontologies. Last, we generated a representative summary from the high-scoring PICO sequences aggregated by study type, publication credibility, and freshness score.

**Results:**

Evaluation of the prognosis quality recognition model using a large dataset of biomedical literature related to intracranial aneurysm showed an accuracy of 95.41% (2562/2686) in terms of recognizing quality articles. The clinical context–aware multiclass classifier outperformed the traditional machine-learning algorithms, including support vector machine, gradient boosted tree, linear regression, K-nearest neighbor, and naïve Bayes, by achieving 93% (16127/17341) accuracy for classifying five categories: aim, population, intervention, results, and outcome. The semantic similarity algorithm achieved a significant Pearson correlation coefficient of 0.61 (0-1 scale) on a well-known BIOSSES dataset (with 100 pair sentences) after semantic enrichment, representing an improvement of 8.9% over baseline Jaccard similarity. Finally, we found a highly positive correlation among the evaluations performed by three domain experts concerning different metrics, suggesting that the automated summarization is satisfactory.

**Conclusions:**

By employing the proposed method Biomed-Summarizer, high accuracy in ATS was achieved, enabling seamless curation of research evidence from the biomedical literature to use for clinical decision-making.

## Introduction

### Background

Automatic text summarization (ATS) is a leading topic in the field of information retrieval research, particularly in the medical and biomedical domains, which offers an efficient solution to access the ever-growing amount of scientific and clinical literature by summarizing the source documents while maintaining their most informative contents [1]. The large quantity of biomedical data now accessible to clinicians makes it challenging to locate the correct data rapidly; thus, automatic summarization can provide highlights particular to clinical needs [2]. Moreover, achieving precise, succinct, and coherent information extraction from credible published biomedical resources to construct a simplified summarization plays an imperative role in clinical decision-making, educating patients, and medical education. At the same time, automating this process will provide better opportunities for users to obtain the most critical points of required clinical knowledge without having to delve into an enormous amount of text, saving hours of searching. It is highly desirable to accurately identify scientifically sound published studies and summarize selected studies against a given type (eg, intervention and prognosis) of clinical query.

The primary requirements in carrying out this task of extracting noteworthy information are the efficiency of the process, contextualization, and precision of retrieved contents. Efficiency pertains to reducing human involvement and achieving the required materials in a timely manner; contextualization refers to the user objective relevant to a clinical task; and precision of contents applies to the correct identification of needed contents from trustworthy resources. Owing to the massive increase in the biomedical research literature, finding relevant, scientifically sound, and fit-to-context information has become more and more challenging for precise automatic text classification and summarization. The field of text classification and ATS is well-explored [3-7]; however, minimal work has been done in a clinically fit-to-context summary of biomedical text [8,9]. Medical text summarization poses a unique set of challenges compared to summarization in other domains [10].

The main limitations of existing medical text summarization are as follows: (a) inability to capture the clinical context while ranking the sentences for the summary; (b) lack of consideration in checking the quality of the documents before performing summarization; (c) inability of identifying context and implicit information present in the biomedical text, which cannot explicitly match with the user query; (d) lack of purpose-driven ranking and selection of passage for the final summary; and (e) nonuse of a large training set for training deep neural network models. To overcome these limitations, this paper introduces a novel framework called Biomed-Summarizer, which provides a quality-aware Patient/Problem, Intervention, Comparison, and Outcome (PICO)-based intelligent and context-enabled summarization of contents of biomedical literature to satisfy the requirements listed above. Unlike the use of traditional features using a bag of words (BoW) approach, in which the text is tokenized into multiple words and each word is given a number representing the frequency of its use [3], more powerful word-embedding techniques have recently been introduced, such as word2vec [11,12] and GloVe [13], to combat the issues of data sparseness and context-overlooking by generating a vector space, typically of several hundred dimensions [14]. The word2vec approach creates vectors at the sentence or document level by employing the sentence2vec or doc2vec model [15]. The Biomed-Summarizer framework uses the Keras tokenizer [16] to generate a contextually rich set of features with a compact number of dimensions to enable semantics-based matching for precise extraction, summarization of contents, and avoiding sparseness. These features are then used as input to our bidirectional long-short term memory (Bi-LSTM) [17] classification model to identify the PICO sequence. PICO was initially proposed for formatting a clinical question. Subsequently, researchers have used the PICO structure for information retrieval and sentence identification in the text of biomedical documents [18-21]. In deciding which sequences to include in the final summary, we considered a comprehensive criterion that provides information on the quality of the study to which that sequence belongs, the relevance of the sequence to the user query, study type, credibility of the publishing venue, and freshness in term of the date of publication.

In summary, the main contributions of this paper are as follows. First, we introduce a novel framework, termed Biomed-Summarizer, for extractive multidocument ATS of biomedical documents. The summary construction is based on PICO elements identified in each record. Additionally, we employ contextual and quality parameters for the selection of a subset of a PICO sequence of sentences to include in the final summary.

Second, for quality recognition, a highly optimized multilayer feed-forward neural network model, multilayer perceptron (MLP), is presented to acquire significantly accurate results. This model offers binary classification to identify the soundness of a study based on data (title and abstract) and metadata (article type, authors, and publishing venue and date) features.

Third, for PICO elements classification, we propose a Bi-LSTM recurrent neural network (RNN) model trained on the vector representation of the text, which significantly boosts the performance compared to conventional machine-learning models such as support vector machine (SVM), logistic regression, decision tree, and naïve Bayes. Unlike previous studies that focused on the detection of PICO elements one-by-one by employing a separate binary classifier for each PICO element, the proposed approach is a multiclassifier model, which classifies PICO sequences simultaneously from any given biomedical study.

Fourth, to accurately extract the PICO sentences to be included in the final summary, we present a novel method of calculating the similarity between the query and medical text using the Jaccard coefficient after semantically enriching the text using medical ontologies.

Finally, we offer a publicly available dataset [22] comprising thousands of abstracts related to intracranial aneurysm (also known as cerebral or brain aneurysm) curated from the biomedical literature for PICO-based classification. Additionally, another open-source dataset [22] is presented for the quality recognition model.

We aimed to achieve these contributions through the precise, succinct, and coherent information extraction from credible published biomedical resources, and to construct a simplified summary containing the most informative contents that provide a review particular to clinical needs.

### Related Works

#### ATS in the Biomedical Domain

Summarization techniques are generally divided into two categories: abstractive and extractive [2,4]. Abstractive summarization methods examine the text and generate a new summarized text as a representative of the original text. In contrast, extractive summarization methods recognize the important part of the text, extract that component, and generate the summary verbatim. Extractive summarization approaches are classified into different categories such as statistical-, topic-, graph-, discourse-, and machine learning–based approaches [5]. Single and multidocument summarization are the two principal categories concerning the number of documents, whereas generic and query-specific are the two main types of summaries [5]; however, another possible criterion can be used to classify these studies into item set–based mining, and classification and ranking. The item set–based mining approach extracts domain concepts in the representative sentences to generate a graph-based summary [1,23,24]. The classification and ranking–based approach first detects key sentences and ranks them according to their importance in the text to produce a plain textual summary. For such a task, some researchers have used statistical features such as term frequency, sentence position, and similarity with the title [25,26], whereas other methods incorporated semantic information extracted from external linguistic resources [26,27].

#### Identification of PICO Elements in Text

Minimal work has been done in the area of PICO-based retrieval of contents from the biomedical literature. The existing studies are categorized based on three aspects: individual PICO element identification [9,18,28], sentence classification [21,29], and question and answer with summarization [9,30]. In a proceeding, the authors presented a process of a PICO corpus at the individual element level and sentence level [31]. A hybrid approach of combining machine learning and rule-based methods was proposed for the identification of PICO sentences and individual elements in successive order [20]. Another study on PICO sentence extraction was carried out with a supervised distance supervision approach that capitalizes on a small labeled dataset to mitigate noise in distantly derived annotations [32]. The authors developed a naïve Bayes–based classifier and reported that it is not sufficient to rely only on the first sentence of each section, particularly when high recall is required [28]. Boudin et al [33] used multiple supervised classification algorithms to detect PICO elements at the sentence level by training data on structured medical abstracts for each PICO element. The results showed that the detection accuracy was better for the Patient/Problem compared to the Intervention and Outcome elements.

#### Quality of Biomedical Studies

Several promising approaches have been explored [34-41] to retrieve high-quality (ie, scientifically sound) studies from the PubMed database. Among these, some methods such as PubMed Clinical Queries rely on Boolean-based strategies using Medical Subject Heading (MeSH) terms and keywords [39,40]. Clinical Queries are often considered as a reference point for assessing the effectiveness of approaches intended to retrieve scientifically sound studies from PubMed. Some methods have also used supervised machine-learning algorithms such as SVM, naïve Bayes, and decision tree to identify scientifically rigorous studies from PubMed [34,37,38,42]. These approaches mainly depend on bibliometric features, semantic features, and MeSH terms. The limitation of approaches that use MeSH terms is the availability, as MeSH terms are added to PubMed citations with an average time gap of 20 to 252 days after an article is published [40,43]. Very recently, a deep-learning approach was used for the task of detecting quality articles in PubMed [40,41]. Deep learning–based approaches have proven to improve accuracy over the existing approaches of machine-learning models, PubMed Clinical Queries search, and McMaster text word search in terms of precision. These approaches were trained on a treatment-related dataset.

#### Sentence Scoring and Ranking for Summarization

Various methods have been proposed to choose what text should be included in the final summary [1,4-8,23,24,44]. The most common method is the frequency-based approach, in which a sentence with a word holding the highest frequency is given more importance [45]. Some studies have found similarities in a sentence with the title of the document [5]. If a sentence encompasses words in the title, the sentence is assigned a score value of 1; otherwise, a score value of 0 is assigned. Another approach commonly followed for the selection of sentences to include in the summary is the cue words technique, in which the cue words are provided by the user [4,6,44]. A score value of 1 is assigned to the sentence if it contains these cue words; otherwise, a score value of 0 is assigned. Sentence position and length are also considered for sentence inclusion in the summary. These techniques are used to score a sentence by linearly combining the individual scores of each method [44]. Based on the scoring, a certain number of sentences with high ranks are picked up to include in the final summary. Recently, deep-learning approaches have been widely used in text summarization [3,46-49]. A neural network–based general framework was proposed for single-document summarization using a hierarchical document encoder and attention-based extractor [48]. For the sentence ranking task of multidocument summarization, a ranking framework was presented using a recursive neural network (R2N2) [49]. A query-focus summarization system called AttSum [46] was proposed that determines ranking based on *both* saliency and relevance in contrast to previous approaches of learning to rank based on *either* relevance or saliency.

The prominent issues with existing approaches are as follows: (a) inability to capture the clinical context while ranking the sentences for creating summarization, (b) omitting the check on the quality of the documents used in the summary, (c) inability of identifying implicit information located in the body text of a study that is not explicitly matched with the user query, (d) lack of purpose-driven ranking and selection of passage for the final summary, and (e) unavailability of a large training set for training deep neural network models. Biomed-Summarizer circumvents all of these limitations.

## Methods

### Overall Design

**Figure 1:**
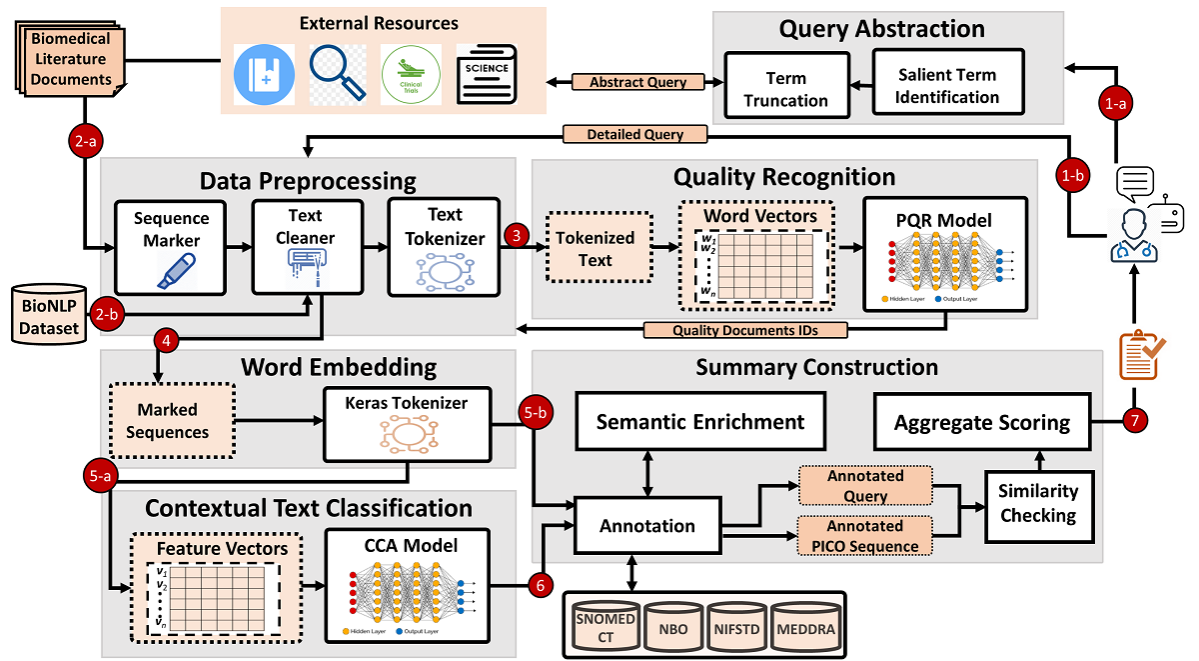
Proposed Biomed-Summarizer architecture with four major components: data preprocessing, quality recognition, context identification, and summary construction. PQR: prognosis quality recognition; CCA: clinical context-aware; PICO: Population/Problem, Intervention, Comparison, Outcome.

Figure 1 shows the architecture of the proposed Biomed-Summarizer framework highlighting four main modules: data preprocessing, quality recognition, contextual text classification, and text summarization. The main tasks in data preprocessing are sequence marker, cleaning, tokenization, vector generation, and feature creation. The quality recognition module identifies the scientific soundness of a study using a feed-forward deep-learning binary classifier that is trained on biomedical data and metadata features. The contextual text classification classifies the PICO sequence of sentences in the selected quality documents using the Bi-LSTM model, an RNN trained on a large set of data acquired from BioNLP [21] and domain-specific Medline abstracts. Lastly, the summarization module generates a summary from the classified PICO sequences of multiple documents using a sequence scoring method consisting of semantic similarity (between a query and a text sequence extracted from a biomedical study), the publishing venue’s credibility, and year of publication.

### Data Preprocessing

Biomed-Summarizer acquires data from two input sources: BioNLP dataset and PubMed. The BioNLP dataset is publicly available, whereas we retrieved the domain-specific abstracts from PubMed. The process used to parse raw abstracts retrieved from the PubMed and BioNLP is described below.

#### Sequence Marker

The task of sequence marker is to parse each raw abstract in the dataset to retrieve the headings based on the keywords listed in the dictionary, as shown in Textbox 1. If a keyword is matched in the abstract, we extract the text (sequence of sentences) under that heading and mark it with the corresponding label. For instance, if a heading “objective” is found in an abstract, label “A” is assigned to the text. The dictionary is based on a previous study [21] with extension of a few more keywords (eg, Patient 1 and Patient 2). This process is repeated for all of the abstracts in the dataset of documents retrieved from PubMed.

**Master dictionary representing keywords that appear in headings in the structured abstracts of biomedical studies.**

dict = {‘A’: ['objective,' 'background,' 'background and objectives,' 'context,' 'background and purpose,'

'purpose,' 'importance,' 'introduction,' 'aim,' 'rationale,' 'goal,' 'context,' 'hypothesis'], 'P': ['population,' 'participant,' 'sample,' 'subject,' 'patient,' 'patient 1,''patient 2'],  'I': ['intervention,' 'diagnosis'], 'O': ['outcome,' 'measure,' 'variable,' 'assessment'], M': ['method,' 'setting,' 'design,' 'material,' 'procedure,' 'process,' 'methodology'], 'R': ['result,' 'finding'], 'C': ['conclusion,' 'implication,' 'discussion,' 'interpretation table']}

We employed the following steps to prepare data for the clinical context–aware (CCA) classification model.

#### Text Cleaning

The process of text cleaning removes special characters, punctuation, stop words, and URLs present in the text. For removing stop words, we used the Python NLKT library [50].

#### Tokenization

This process splits the sentences retrieved after the text cleaning process into individual tokens. We used the Keras tokenizer [16] to create a list of tokens for each biomedical paragraph.

#### Vector Generation

We considered a maximum of 50,000 words for each paragraph and generated a 250-dimension vector representation using Keras text [16] to sequence functionality. We made sure that each vector length is the same.

As a result of the above steps, we obtained a vector representation of each paragraph, which is then used for training and testing of the CCA classification model. An example of text sequences and corresponding class labels is shown in Table 1. It is important to mention that we do not require a process of sequence marking on BioNLP data because these sentences are premarked.

**Table 1:** Example sequence of sentences for an assigned category of Aim, Population, Methods, Interventions, Results, Conclusion, and Outcomes.

Sequence	Category
The aim of the present study was to evaluate whether the Anterior communicating artery (A com) aneurysms behave differently from the aneurysms located elsewhere with respect to size being a rupture risk. To this end, we examined the clinical data of ruptured A com aneurysms and analyzed other morphological parameters, including size parameter, providing adequate data for predicting rupture risk of the A com aneurysms.	Aim (A)
Between January 2010 and December 2015, a total of 130 consecutive patients at our institution with the A com aneurysms-86 ruptured and 44 unruptured-were included in this study. The ruptured group included 43 females (50%) and 43 males (50%) with the mean age of 56 years (range, 34-83 years). The unruptured group included 23 females (52%) and 21 males (48%) with the mean age of 62 years (range, 28-80 years). All patients underwent either digital subtraction angiography or 3-dimensional computed tomography angiography. The exclusion criteria for this study were the patients with fusiform, traumatic, or mycotic aneurysm. There were preexisting known risk factors, such as hypertension in 73 patients, who required antihypertensive medication; other risk factors included diabetes mellitus (9 patients), coronary heart disease (9 patients), previous cerebral stroke (18 patients), and end-stage renal disease (3 patients) in the ruptured group. In the unruptured group, 38 patients had hypertension, 4 had diabetes mellitus, 5 had coronary heart disease, 10 had a previous cerebral stroke, and 2 had end-stage renal disease.	Population (P)
Four intracranial aneurysms cases were selected for this study. Using CT angiography images, the rapid prototyping process was completed using a polyjet technology machine. The size and morphology of the prototypes were compared to brain digital subtraction arteriography of the same patients.	Methods (M)
After patients underwent dural puncture in the sitting position at L3-L4or L4-L5, 0.5% hyperbaric bupivacaine was injected over two minutes: group S7.5 received 1.5 mL, Group S5 received 1.0 mL, and group S4 received 0.8 mL. interventions after sitting for 10 minutes, patients were positioned for surgery.	Intervention (I)
The ruptured group consisted of 9 very small (<2 mm), 38 small (2-4 mm), 32 medium (4-10 mm), and 7 large (>10 mm) aneurysms; the unruptured group consisted of 2 very small, 16 small, 25 medium, and one large aneurysms. There were 73 ruptured aneurysms with small necks and 13 with wide necks (neck size>4 mm), and 34 unruptured aneurysms with small necks and 10 with wide necks.	Results (R)
The method which we develop here could become surgical planning for intracranial aneurysm treatment in the clinical workflow.	Conclusion (C)
The prevailing view is that larger aneurysms have a greater risk of rupture. Predicting the risk of aneurysmal rupture, especially for aneurysms with a relatively small diameter, continues to be a topic of discourse. In fact, the majority of previous large-scale studies have used the maximum size of aneurysms as a predictor of aneurysm rupture.	Outcome (O)

### Quality Recognition Model

**Figure 2:**
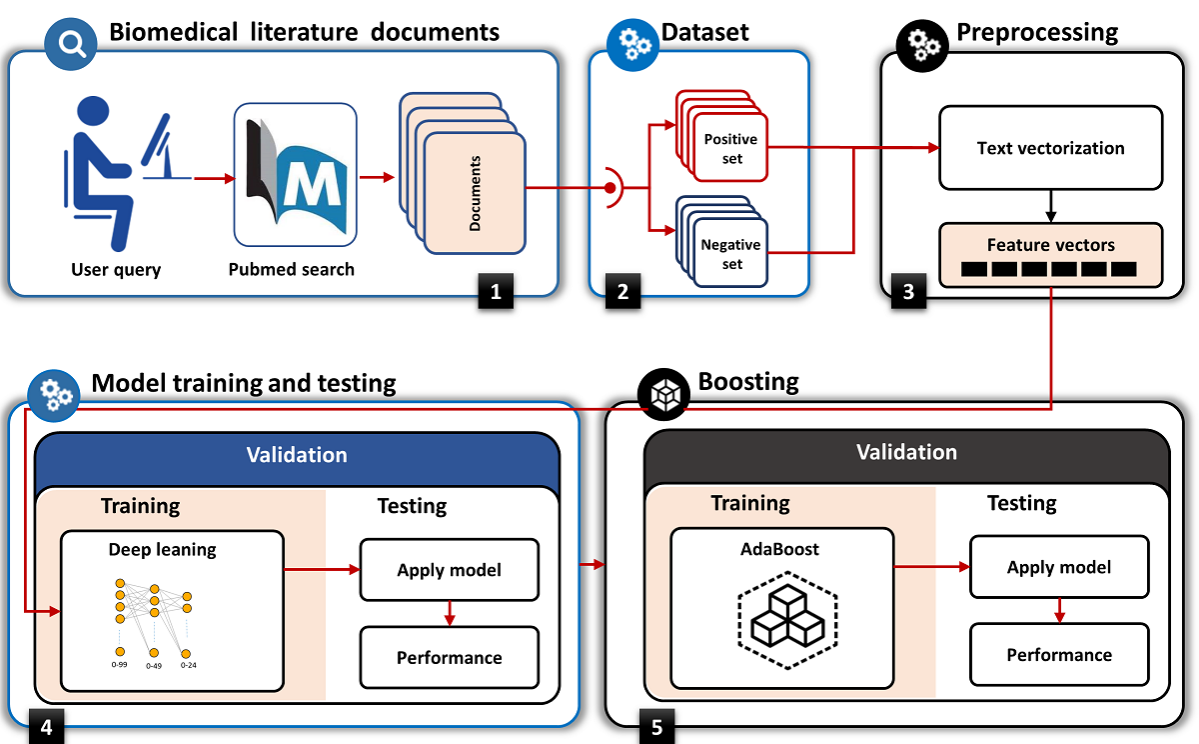
Process steps of proposed prognosis quality recognition (PQR) model training and testing.

The proposed model, as shown in Figure 2, comprises multilayer feed-forwarded neural networks as a so-called MLP. The MLP is further optimized with an ensemble method using adaptive boosting (AdaBoost). The final AdaBoost-MLP, termed the prognosis quality recognition (PQR) model, was trained on a dataset acquired automatically through PubMed searches based on the following two criteria: selecting the “Clinical Query prognosis” filter and choosing scope as “narrow.” To build and evaluate the model, we performed the steps involved in the preparation of a dataset for training the deep-learning models, followed by training and tuning the deep-learning models. Additionally, we compared the performance of the proposed model with those of shallow machine-learning models in terms of precision and recall.

For training and testing the PQR model, we collected a dataset consisting of a total of 2686 Medline abstracts, 697 of which were considered to be positive studies (ie, scientifically sound). To retrieve positive studies, we used the interface of Clinical Queries [33]. To retrieve negative studies (ie, not scientifically sound), we used the interface of PubMed and retrieved studies that were not included in the positive set.

### Training and Tuning the Deep-Learning Model

The PQR model is trained using five textual features, including two data features (title and abstract) and three metadata features (article type, publishing journal, and authors). The data features were preprocessed through applying the steps described above. The model consists of 5 layers with 3 hidden layers of size 100, 50, and 25, respectively. The input layers take the BoW vectors generated from data features. The “Maxout” activation function was used with 10 epochs. The data were split into a 70:30 ratio for training and testing.

### Comparison with Shallow Machine-Learning Models

To identify the best performer on our dataset, we compared a set of machine-learning algorithms, including SVM, naïve Bayes, k-nearest neighbor, and decision tree. These algorithms were tested in other studies [35,39] for addressing similar problems.

### CCA Classification Model

The CCA classification model aims to develop a multiclass classifier that can accurately classify text from selected quality documents (given by the quality model) into one of the following 7 classes: Aim, Methods, Population, Intervention, Results, Outcome, and Conclusion. We then merged Methods and Population into a single category (P) and Outcome and Conclusion into a single category (O) because of their strong correlation found in the text. Although our focus was to utilize PICO classes in the summarization task, we retained the other categories to enable additional non-PICO clinical applications such as summarization for medical education and deriving rules from clinical trials for developing the knowledge base of a clinical decision support system.

PICO is a well-known terminology in evidence-based medicine. Our proposed CCA classifier incorporates the Patient/Problem, Intervention, and Outcome PICO components in addition to two more classes, Aim and Results. PICO detection is a sequential sentence classification task rather than a single sentence problem; therefore, the sequence of sentences is jointly predicted. In this way, more complete context is captured from multiple sentences, which then improves the classification accuracy of predicting the current sentence. The steps followed to build and evaluate the model were: (a) preparation of a dataset for training the deep-learning model, (b) training and tuning the deep-learning model, and (c) comparison with traditional machine-learning models in terms of precision and recall.

### Model Building

The neural network model is heavily used in text processing due to the ability to process arbitrary-length sequences. RNNs are immensely popular in multiclass text classification. To build and evaluate the classification model, we employed the Bi-LSTM model as a type of RNN. This model preserves the long-term dependency of the text and is one of the most popular models in text classification.

**Figure 3:**
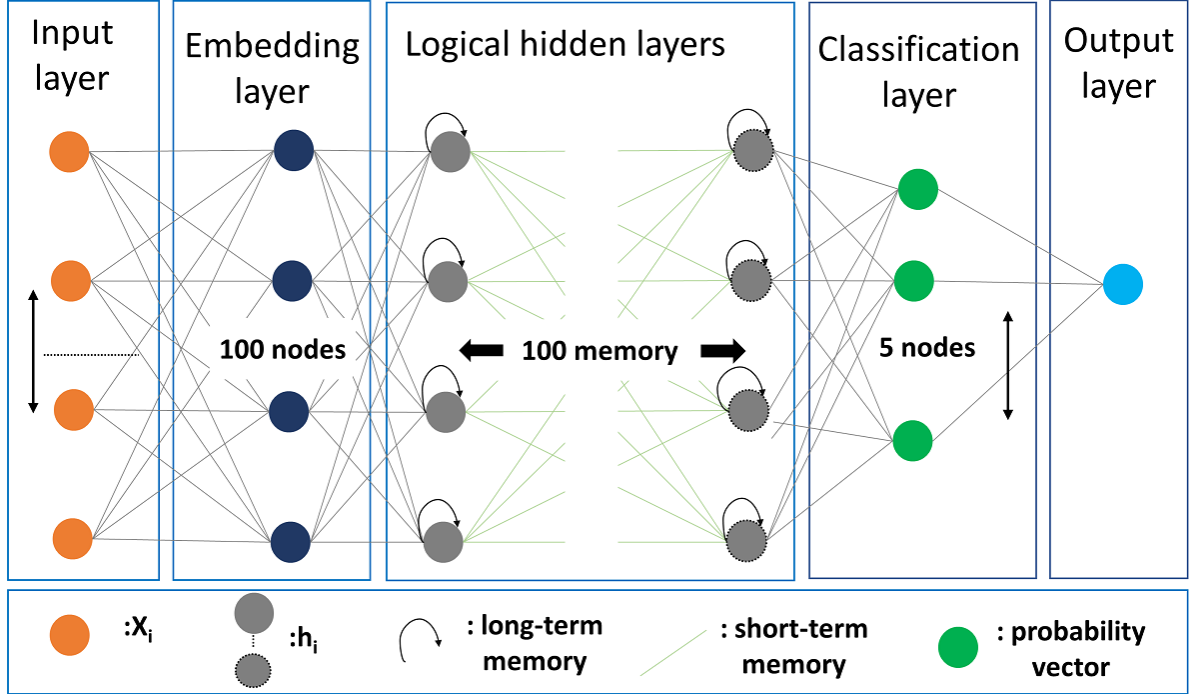
Clinical context–aware (CCA) classifier trained on 250-dimension feature vectors, 100 nodes at the embedding layer, 100 memory units of the long short-term memory (LSTM) layer logical hidden layers, and 5 classification nodes.

As shown in Figure 3, the input layer consists of 250 dimensions showing the numeric features created using the Keras tokenizer, embedding layer, LSTM logical hidden layers, classification layer, and output layer. In brief, the trained CCA model has the following features: (1) an initial training dataset comprising 173,401 records, 90% (n=156,060) of which were used for training and 10% (n=17,341) of which were used for testing; (2) the first hidden layer is the embedding layer that uses 100-dimension vectors to represent each paragraph; (3) the next layer is the LSTM layer with 100 memory units, and the recurrent dropout rate was set to 0.2; (4) the next layer is the dense layer with 5 nodes, and we used the SoftMax activation function for multiclassification, and categorical_crossentropy as a loss function; (5) the training dataset is further divided into two parts, 10% of which was used to validate the category and minimize the loss function, resulting in 140,454 records used for training and 15,606 used for validation; (6) the model was then trained across 5 epochs with a minibatch size of 64.

### ATS

For the ATS task, we developed a sentence-scoring mechanism based on a multifeatured matrix, consisting of 4 features (relevance, study type, venue credibility, and freshness), where each feature was learned with a specific method as explained in the following sections.

First, an individual score value is assigned to each feature, which is then aggregated in the final column as a final score using the formula in Equation 1:

Aggregate Score = *βR_score_* + *γ*(*ST_rank_* + *VC_score_* + *f_score_*) **(1)**,

where *R_score_* is the relevance score, *ST_rank_* is the study type rank, *VC_score_* is the publishing venue credibility score, *f_score_* is the freshness score, and *β*=70 and *γ*=10 are the scaling constants to keep the aggregate score in the range of 0 to 100.

### Relevance Score

For the relevance score, we developed a semantic similarity algorithm, Jaccard similarity with semantic enrichments (JS^2^E) as a 6-step algorithm that uses the BioPortal application programming interface to access biomedical ontologies (SNOMED CT, MedDRA, NBO, NIFSTD) for obtaining semantic enrichment and the Jaccard similarity metric to find similarities between two texts.

In step 1, an individual sequence of text is obtained from the query, which is preprocessed to remove the special characters, separate characters, and different formatting tags. In step 2, the preprocessed text is annotated using BioPortal. In step 3, each token is enriched using “definition,” “synonyms,” and “prefLabel” relations from selected ontologies. In step 4, the annotations of text are retrieved using the “definition” relationship, along with the preprocessing (step 1) and annotation (step 2) procedures. In step 5, the annotated tokens received in step 2 are combined with the “synonyms” and “preflabel” obtained in step 3 and the annotated tokens received in step 4, and the data structure of metatokens is constructed. Finally, in step 6, the Jaccard similarity between the metatokens of the text sequence and the query is calculated using Equation 2:

*R_score_*=*S_m_* ∩ *Q_m_*/*S_m_*∪ *Q_m_***(2)**,

where *S_m_* are the metaset tokens of the text sequence in a document and *Q_m_* are the metaset tokens of the query.

### Study Type

The study type plays a vital role in proving a study’s usefulness for a user concerning a clinical task. For instance, if a surgeon wants to look for advances in successful surgical treatments, randomized control trials or a meta-analysis of randomized controlled trials will be the priority. The priority will change if the user is interested in prognosis-related studies. Study types can be used to grade evidence concerning quality and other elements [34,51,52]. To find the priority of different study types in the category of prognosis, we conducted a questionnaire-based survey among domain experts who were asked to assign a score value for each study type. An example of a filled-in questionnaire by a physician is provided in Multimedia Appendix 1.

The final rank value is then learned from the average value of rank values assigned by the domain experts, as shown in Equation 3.


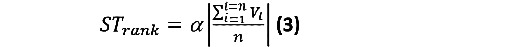


where *V_i_* represents the rank values assigned to a study type by each domain expert, *n* is the total number of domain experts that participated in the questionnaire, and α=0.1 is a scaling constant to keep the final score value in the range of 0 to 1.

### Venue Credibility

The credibility of a study publishing venue, including journals, proceedings, and books, is also an important parameter; however, it is more of a subjective matter. Therefore, it is necessary to consult with the stakeholders of the service. For this study, we sought to obtain a list of valuable publishing venues. We involved resident doctors to rank various venues concerning their credibility. An example of a filled questionnaire by a physician is provided in Multimedia Appendix 1. The final credibility score was determined from the average value of rank values assigned by the domain experts, as shown in Equation 4:


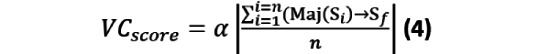


where *S_i_* is the initial rank assigned by the domain experts, *S_f_* is the mapped score obtained through mappings {1→10, 2→9, 3→8, 4→7, 5→6, and 6→5}, and α=0.1 is a scaling constant to keep the final score value in the range of 0 to 1. We applied the majority vote method before using the mapping function.

### Freshness

Freshness represents the date of publication of a study, which is useful to consider to keep up with the advancement in a domain. We included this attribute in summarization with a higher rank assigned to more recent studies, following the less current studies, as described in Table 2.

**Table 2:** Assigned weights for research study year of publication.

Year of publication	Rank
Previous 1-5 years	1
Previous 6-10 years	2
Previous 11-15 years	3
Other	4

The final score was calculated according to Equation 5:

*F_score_*=α(*R_i_*→*R_f_*) **(5)**,

where *R_i_* is the initial value assigned to each year obtained from Table 2, *R_f_* is the mapped score obtained through mappings {1→10, 2→9, 3→8, and 4→7}, and α=0.1 is a scaling constant to keep the final score value in the range of 0 to 1.

### Text Selection for Summary

The aggregate score calculated using Equation 1 provides the final rank values for a text sequence. Two types of summary structures can be generated: (1) a PICO-based summary, in which we select the top *k* text sequences in each part of PICO out of a total *n* number of sentences to be included in the final summary as |*n*/*k*| sentences from the Patient/Population component, |*n*/*k*| sentences from the Intervention component, and |*n*/*k*| from the Outcome component; and (2) a non-PICO-based summary, where we select the top *k* text sequences without considering their classification.

### Summary Presentation

Our proposed model of automatic summarization can generate a summary for a single document or multiple documents simultaneously. For summaries of a single document, the selected set of representative PICO sequences is arranged in a template of Population/Problem, Intervention, and Outcome, as shown in Textbox 2. The templates are displayed according to the number of documents retained after a quality check. For instance, if we have a set of 5 studies retrieved against a user query, our algorithm will generate 5 individual summaries presented in the order of the most recent document summary followed by the others.

**Example summary of a biomedical document represented with the Patient/Problem (P), Intervention (I), and Outcome (O) sequence.**

P: One hundred and fifty patients …duration at a frequency of at least once per week

I: After patients underwent dural puncture … patients were positioned for surgery

O: Number of follow-up appointments attended … occurrence of secondary ocular hypertension

In the case of a multidocument summary, our algorithm first identifies PICO sequences in all documents, finds the score for each sequence, selects the highest-scoring sequence in each category, and concatenates all scored sequences to obtain a combined summary of each type. In this case, the sequences included in the summary may or may not belong to a single document; however, each sequence is linked to its corresponding documents for audit, transparency, and education.

### Example Case: Step-by-Step Scenario of Summary Generation

**Figure 4:**
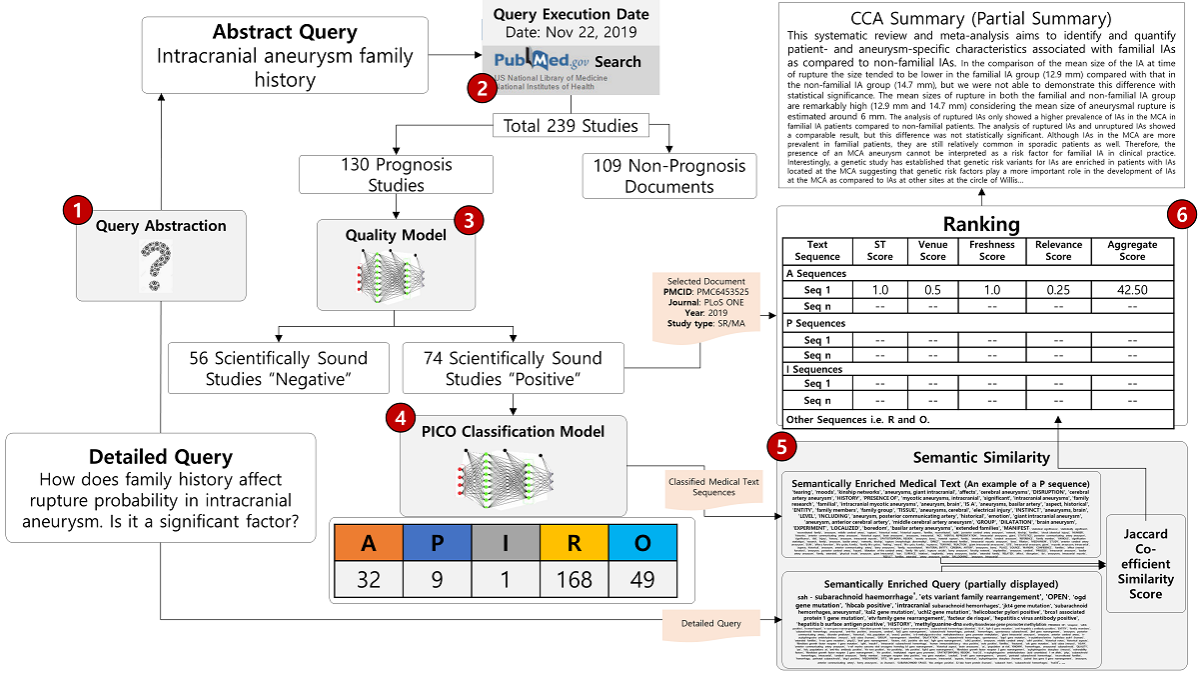
Step-by-step scenario of query execution, retrieval of documents, quality checking, clinical context-aware (CCA) classification, semantic similarity, ranking, and summary creation. A: Aim; P: Population/Patients/Problem; I: Intervention; R: Results; O: Outcome; PICO: Patient/Problem, Intervention, Comparison, Outcome.

To clarify the steps of the proposed Biomed-Summarizer framework, we take the following example query: “How does family history affect rupture probability in intracranial aneurysms; is it a significant factor?“ (Abstract Query-Intracranial aneurysm family history). As shown in Figure 4, the user query was first abstracted from a detailed user query to increase the recall of retrieving studies. Second, the query was run on the PubMed search service, which returned a total of 239 studies, 130 of which were prognosis studies. Third, these studies were run through a quality model, “PQM,” which identified 74 studies as scientifically sound and the rest were filtered out. Fourth, the set of 74 studies was given to the PICO classification model in the form of text sequences, which were classified into five classes: Aim (32), Patients (9), Intervention (1), Results (168), and Outcome (49). Fifth, the PICO text sequences and the detailed query were passed through semantic similarity, in which the texts were first enriched semantically using medical ontologies, and the similarity score was calculated using the Jaccard coefficient. Sixth, the documents were ranked according to the accumulated score of four parameters: relevance score of the query and text, study type, venue’s credibility score, and freshness score. Finally, the required summary is created and presented to the user.

## Results

### Dataset Preparation

To evaluate the performance of the proposed framework, we prepared two different datasets to test the proposed Biomed Summarizer framework. One dataset was used for the development and testing of the PQR model (D1), whereas the other was used for the development and testing of the CCA model (D2). The preparation protocols of both datasets are outlined in Table 3. These datasets are available to the research community via a GitHub link [22].

**Table 3:** Dataset preparation protocols.

Preparation Protocol	PQR^a^ dataset (D1)	CCA^b^ dataset (D2)
Description	This dataset was created for the quality assessment of biomedical studies related to the prognosis of brain aneurysm.	This dataset was curated for the use of PICO^c^ sequence classification. The final dataset was specific to the prognosis of brain aneurysm.
Purpose	To select only published documents that are scientifically rigorous for final summarization.	To identify a sentence or a group of sentences for discovering the clinical context in terms of population, intervention, and outcomes.
Methods	The manual preparation of the dataset is a cumbersome job, and thus AI^d^ models were used. For development of an AI model, a massive set of annotated documents is needed. Annotation is a tedious job; therefore, PubMed Clinical Queries (narrow) as a surrogate were used to obtain scientifically rigorous studies.	N/A^e^
Data sources	PubMed Database (for positive studies, the “Narrow[filter]” parameter was enabled).	First, we collected a publicly available dataset, BioNLP 2018 [21], which was classified based on the PICO sequence in addition to “Method” and “Results” elements. To increase the dataset size, we added more sentences related to brain aneurysm created from Medline abstracts retrieved using the NCBI^f^ PubMed service Biopython Entrez library [53].
Query	The term “(Prognosis/Narrow[filter]) AND (intracranial aneurysm)” was used as a query string.	The term “Intracranial aneurysm” (along with its synonyms “cerebral aneurysm” and “brain aneurysm”) were used as a query string.
Size	2686 documents, including 697 positive (ie, scientifically rigorous) records	A total of 173,000 PICO sequences (131,000 BioNLP+42,000 Brain Aneurysm) were included in the dataset.
Inclusion/exclusion	Only studies that were relevant and passed the criteria to be “Prognosis/Narrow[filter]” were included in the positive set. The other relevant studies not in the positive set were included in the negative set. All other studies were excluded from the final dataset.	Only structured abstracts identified with at least one of the PICO elements were considered to extract the text sequence.
Study types	RCTs^g^, systematic reviews, and meta-analysis of RCTs were given more importance.	RCTs, systematic reviews, and meta-analysis of RCTs were given more importance.

^a^PQR: prognosis quality recognition.

^b^CCA: clinical context–aware.

^c^PICO: Patient/Problem, Intervention, Comparison, Outcome.

^d^AI: artificial intelligence.

^e^N/A: not applicable.

^f^NCBI: National Center of Biotechnology Information.

^g^RCT: randomized controlled trial.

### Experimental Setup

To evaluate the CCA and PQR classification models, we performed experiments on a 64-bit Windows operating system with an Intel Core i5 CPU, 3.20-Hz processor with 8-GB memory using dataset D1 and D2, respectively. The experiment was performed using RapidMiner studio [54] to train and test the PQR models, whereas the python deep learning library Keras was used for the CCA classification model [55].

### PQR Model

The aim of this experiment was to quantify the comparative analysis of the proposed deep-learning model with other machine-learning models. Using the default settings of RapidMiner, the SVM model followed the kernel type “dot,” kernel cache of 200, and complexity constant *c* of 0.0; the DT criterion was “gain_ratio” with a maximum depth of 10; the *k* of the k-nearest neighbor was 5 with the mixed measure of “MixedEuclideanDistance.” The comparison of the performance of these models with that of a deep-learning model was assessed in terms of the F1-score, accuracy, and area under the curve (AUC) value, as shown in Table 4.

We tuned the hyperparameters of the MLP by varying the hidden layers, size of layers, activation function, and epochs, and obtained varied results as shown in Table 5.

**Table 4:** Comparative results of the deep-learning model with shallow machine-learning models.

Algorithm	F1-score	Accuracy	AUC^a^
Naïve Bayes	90.83	87.47	0.987
Decision tree	85.10	74.07	0.50
k-nearest neighbor	46.53	48.39	0.829
General linear model	89.34	82.38	0.904
Support vector machine	86.96	77.79	0.983
Deep learning (MLP^b^)	93.17	90.20	0.967

^a^AUC: area under the receiver operating characteristic curve.

^b^MLP: multilayer perceptron.

**Table 5:** Results of multilayer perceptron with varied hyperparameter settings.

Hidden layers (n)	Hidden layer size	BoW^a^	Activation	Epochs (n)	Recall	Precision	F1-score	Accuracy	AUC^b^
2	50, 50	No	Rectifier	10	90.28	96.25	93.17	90.2	0.967
3	100, 50, 25	No	Rectifier	10	93.47	96.71	95.06	92.8	0.969
3	100, 50, 25	No	Maxout	10	96.82	96.01	96.41	94.67	0.976
3	100, 50, 25	No	Maxout with Dropout	10	98.16	93.61	95.83	93.67	0.963
3	100, 50, 25	No	Tanh	10	90.62	97.65	94.00	91.44	0.978
3	100, 50, 25	Yes	Rectifier	10	93.47	98.24	95.80	93.92	0.999
3	100, 50, 25	Yes	Maxout	10	94.47	97.41	95.92	94.04	0.977
3	50, 50, 50	No	Rectifier	10	87.77	96.86	92.09	88.83	0.958
3	200, 100, 50	No	Rectifier	10	92.96	96.86	94.87	92.56	0.975
4	200, 100, 50, 25	No	Rectifier	10	93.63	96.05	94.82	92.43	0.973

^a^BoW: bag of words.

^b^AUC: area under the receiver operating characteristic curve.

As highlighted in Table 5, the highest accuracy and F1-score were obtained with the setting of 3 hidden layers consisting of 100, 50, and 25 neurons, respectively. The activation function was set to Maxout, and the model was trained on 10 epochs. Finally, we boosted the performance of the selected model with an ensemble approach using AdaBoost. The results of the optimized version of the proposed model are shown in Table 6, demonstrating an F1-score of about 97% and an accuracy of 95.41% with an AUC of 0.999.

**Table 6:** Ensembling of deep-learning models.

Boosting Model	Recall	Precision	F1-score	Accuracy	AUC^a^
Ensemble voting (MLP^b^, DT^c^, NB^d^)	95.81	97.28	96.54	94.91	0.955
Proposed model (AdaBoost^e^-MLP)	97.99	95.9	96.93	95.41	0.999

^a^AUC: area under the receiver operating characteristic curve.

^b^MLP: multilayer perceptron.

^c^DT: decision tree.

^d^NB: naïve Bayes.

^e^AdaBoost: adaptive boosting.

### CCA Model

We experimented with the combination of dataset D1 described above. The classification model results are shown in Table 7.

The individual decision class performance of the proposed model is reported in Table 8. We found high F1-score values (≥80) for classes with top support (Aim, Outcome, Results, Population) as compared to the scores of the minor classes, which indicated that the dataset size needed to be increased for each of these minor classes to obtain a higher F1-score.

**Table 7:** Comparative results of deep learning with traditional machine-learning models.

Model	Recall	Precision	F1-score	Accuracy
Logistic Regression	0.42	0.34	0.36	0.42
AdaBoost^a^	0.49	0.48	0.46	0.49
Gradient Boost	0.50	0.59	0.45	0.50
ANN^b^	0.29	0.08	0.13	0.29
kNN^c^	0.35	0.36	0.37	0.35
Proposed Bi-LSTM^d^ model	0.93	0.94	0.94	0.93

^a^AdaBoost: adaptive boosting.

^b^ANN: artificial neural network.

^c^KNN: k-nearest neighbor.

^d^Bi-LSTM: bidirectional long-short term memory.

**Table 8:** Precision, recall, F1-score, and support for individual classes of the proposed deep-learning model.

Class	Precision	Recall	F1-score	Support
Aim	0.94	0.95	0.95	3133
Intervention	0.84	0.94	0.89	1238
Outcome	0.96	0.94	0.95	5036
Result	0.96	0.94	0.95	4852
Population	0.94	0.95	0.94	3082

### Proposed Semantic Similarity Algorithm (JS^2^E)

We measured the correlation strength based on the standard guideline of the Pearson correlation coefficient in the biomedical domain [20]. We evaluated different similarity approaches, including string similarity (Jaccard), term frequency (count vectorizer), and pretrained word embedding using GloVe, Google word2vec, and fastText. The results showed that semantic enrichment is crucial to find the similarity between the texts because it significantly increases the size of the token set of texts by including synonyms, definitions, and prefLabel from ontologies. We tested this method on a well-known biomedical dataset, BIOSSES [56], which contained 100 pairs of sentences manually annotated by the experts. As shown in Table 9, the Pearson correlation coefficient after semantic enrichment increased by 8.9% relative to that of the best-performing Jaccard similarity. Concerning the pretrained word-embedding model GloVe, word2vec (Google), and fastText (Facebook), the correlation improved by 41.8%, 60.5%, and 17.3%, respectively.

**Table 9:** Comparison of similarity approaches.

Methods	Pearson correlation coefficient (0.0-1.0)
Jaccard similarity	0.56
Cosine similarity (Count Vectorizer)	0.54
GloVe Embedding	0.43
Word2Vec (Google)	0.38
fastText (Facebook)	0.52
Jaccard Similarity after semantic enrichment (JS^2^E)	0.61

### Expert Evaluation of Candidate and Reference Summaries

The PICO-based summary obtained after classification and performing JS^2^E was then compared with the classical summary obtained using JS^2^E without classification. We considered the JS^2^E summary without classification as a baseline summary. Evaluation and comparison of two summaries were performed by three independent evaluators and scored between 0 and 5 on the following three metrics: summary relevance to the inbound query (M1); Aim, Population, Intervention, Results, and Outcome classification representation in the summary (M2); and model summary better than the baseline summary (M3).

Table 10 shows the Pearson correlation coefficients of the scores of each evaluator (A, B, and C) concerning the average scores of the remaining two evaluators for each evaluation metric (M1, M2, and M3). There was a strong association among the scores of each evaluator concerning each metric, suggesting that automated summarization performed best on all three parameters. The lowest correlation coefficient was 0.728, which is still considered to be high on the correlation scale.

The distribution of the scores by each of the evaluators concerning each metric is described in Table 11. The distribution suggests that there are sufficient instances for each of the scores in the evaluation dataset.

**Table 10:** Correlation matrix (Pearson correlation coefficients) of similarity approaches among evaluators for summaries according to the three metrics.

Metric	Evaluator A	Evaluator B	Evaluator C
M1^a^	0.728	0.767	0.837
M2^b^	0.826	0.924	0.841
M3^c^	0.772	0.843	0.804

^a^M1: summary relevance to the inbound query.

^b^M2: aim, population, intervention, results, and outcome classification representation in the summary.

^c^M3: model summary better than the baseline summary.

**Table 11:** Frequency distribution of scores with respect to each metric by all evaluators.

Metric	Score	Frequency
M1^a^	2	4
M1	3	10
M1	4	12
M1	5	4
M2^b^	2	3
M2	3	10
M2	4	10
M2	5	7
M3^c^	3	12
M3	4	16
M3	5	2

^a^M1: summary relevance to the inbound query.

^b^M2: aim, population, intervention, results, and outcome classification representation in the summary.

^c^M3: model summary better than the baseline summary.

## Discussion

### Principal Findings

The proposed Biomed-Summarizer framework generates extractive summaries for single and multiple documents with acceptable accuracy. The evaluation results signify that the proposed framework performs significantly better than existing approaches, which was also evident from the correlation matrix generated by comparing the candidate and reference summaries obtained for each defined parameter. The PQR model trained on a large dataset of biomedical literature of intracranial aneurysm showed an accuracy of 95.41% in terms of recognizing quality articles. The CCA multiclass classification model outperformed the traditional machine-learning algorithms and achieved 93% accuracy for classifying five categories: Aim, Population, Intervention, Results, and Outcome. The semantic similarity algorithm demonstrated a significant Pearson correlation coefficient of 0.61 (on a 0-1 scale) from a well-known BIOSSES dataset after semantic enrichment, representing an improvement of 8.9% over the baseline Jaccard similarity score.

An accurate clinical summarization is expected to revolutionize the field of domain-specific knowledge graphs [57] and clinical decision support systems. For example, an individual PICO-extracted element from a biomedical text could be represented as a relationship in a knowledge graph, which could then be used for various clinical applications or could be directly supplied to clinical decision support systems for physicians to link to internal data-driven instances. The importance of linking external evidence (extracted from the biomedical literature) to internal evidence is important when the internal data are unable to capture all critical risk factors. One of our motivations was to extract the evidence from a PICO-based summarization of documents relevant to intracranial aneurysm (also known as cerebral or brain aneurysm). Since neither decision support systems nor knowledge graphs providing external evidence for complex neurological diseases such as intracranial aneurysm exist, there is a need to achieve automated summarization for such conditions to enhance translational research and clinical decision making synergistically. The proposed automated summarization framework will be pivotal to develop a hybrid machine-learning and knowledge-based clinical decision support system, termed NeuroAssist [58], which aims to predict aneurysmal rupture (ie, subarachnoid hemorrhage). The other possible application areas of the proposed framework include automation of writing systematic reviews over a set of published clinical trials, extraction of evidence for evidence-based medicine, precision medicine, and development of clinical assistants/chatbots.

The current work in the biomedical domain is mainly focused on issues of concept- or sentence-based relevance without relating a concept or sentence to a clinical context. Although the sentence-based classification approach is well-regarded for ATS, relying on only the relevance of a sentence without capturing its clinical context and semantics may lead to a clinically undesirable summary. Some of the summarization work focuses on creating summaries from abstracts only, which may result in low recall due to missing an important sequence of text that exists only in the body of the document. In addition, in previous works, the dataset used for training and testing [29] contained only 1000 abstracts, which is not sufficient for a deep-learning model to be generalized. Recently, a new dataset was published in the BioNLP 2018 proceedings [21], but it does not consider the quality evaluation of the source documents used for extracting the text for summarization. Therefore, we curated a dataset comprising over tens of thousands of abstracts from Medline and combined it with the BioNLP dataset.

PICO-based ATS remains an unexplored area; however, work has been done on individual aspects such as PICO elements identification in the text [21,29,30], quality recognition of therapy-related biomedical studies [40,41], and sentence-based summarization without PICO and quality evaluation [3,32]. To the best of our knowledge, the proposed Biomed-Summarizer framework for biomedical ATS is the first of its kind to use a quality recognition model, PICO-based classification using LSTM-based recurrent deep neural network model for key sentence identification trained on contextual vectors, and the JS^2^E algorithm for semantic matching of the user query and PICO text sequences.

The proposed approach offers a few potential benefits compared to existing methods. First, unlike traditional machine-learning approaches that depend on features that are well-structured and less noisy, deep learning deals well with an unstructured noisy text. Therefore, our deep-learning model can be reused for domains using data of the same nature. Additionally, the accuracy of the proposed deep-learning model is expected to increase further if the volume of the dataset is extended. Second, our approach considers the quality evaluation of the documents being used in summarization in addition to publishing venue credibility, which offers two-fold benefits: it enhances the confidence of physicians on the system-generated summary and has applicability in real clinical settings, and, because it filters out the documents with the lowest quality from the retrieval set, the computational time of summary creation is reduced with respect to checking the similarity of the text with the query. Third, our proposed model is based on the PICO structure, which provides additional semantics as compared to a non-PICO approach in terms of understanding the problem, interventions, and outcomes.

Traditional approaches are not cognizant of capturing the clinical context; therefore, the resultant summary includes sentences based on a high similarity score and sentence position, and is therefore less clinically effective. Textbox 3 shows an example of a summary generated by the proposed method considering the clinical context; sequence 1 represents the aim of the study, sequence 2 represents the patient population included in the study, and sequence 3 represents the outcome of the study. In contrast, the conventional method selects the top three sequences based on high relevancy but misses the clinical context.

**Effectiveness of Biomed-Summarizer in terms of clinical significance.**

**Conventional Method**

Sequence 1: The prevailing view is that larger aneurysms have a greater risk of rupture. Predicting the risk of aneurysmal rupture, especially for aneurysms with a relatively small diameter....

Sequence 2: Alongside with the posterior communicating cerebral artery and middle cerebral artery of bifurcation, the anterior communicating cerebral artery (A com) is one ….

Sequence 3: The A com artery is known to be one of the common sites of aneurysm rupture. Given the diversity of geometry, the difference of the dominance of A1 arteries ….

**Proposed Method**

Sequence 1: The aim of the present study was to evaluate whether the A com aneurysms behave differently from the aneurysms located elsewhere with respect to size being a rupture risk ….

Sequence 2: Between January 2010 and December 2015, a total of 130 consecutive patients at our institution with the A com aneurysms-86 ruptured and 44 unruptured-were included in this study ….

Sequence 3: The prevailing view is that larger aneurysms have a greater risk of rupture. Predicting the risk of aneurysmal rupture, especially for aneurysms with a relatively small diameter….

### Limitations and Future Directions

The training datasets could be reviewed for the noise generated during the creation of automated annotation to obtain even more accurate results in PQR and CCA models. The current summary is a textual summary that contains sentences in the original form as they are presented in the source documents without any further processing to extract statistical information for an easy catch up of the contents.

### Conclusion

Compared to traditional approaches, state-of-the-art deep neural network models can achieve high accuracy for an ATS task when trained on nonsparse semantically enriched features. Additionally, the automated pipeline for seeking research evidence can enable the seamless curation of voluminous biomedical literature to use in clinical decisions. By designing the proposed Biomed-Summarizer framework, we employed a set of methods for information extraction from credible published biomedical resources to construct a simplified summary that is precise, relevant, and contextually suited to clinical needs. We designed the framework to provide openness for other researchers to use, extend, or even make use of a subpart of it and extend for designing their own systems and services. Alongside the framework, we created a specialized dataset containing PICO elements and a few other text sequences such as Aim, Method, and Result for researchers to use in their experiments in the domain of brain aneurysm. The PICO dataset was extended using a custom data-mining process by incorporating the rigorous text processing techniques on PubMed research documents. This method can be further used to create a PICO dataset in other related biomedical domains by obtaining related research papers from PubMed. The evaluation results signify that the proposed Biomed-Summarizer framework performs significantly better than existing approaches.

## Appendix

Multimedia Appendix 1 Survey questionnaire.

## Abbreviations

AdaBoost: adaptive boosting
ATS: automatic text summarization
AUC: area under the receiver operating characteristic curve
Bi-LSTM: bidirectional long-short term memory
BoW: bag of words
CCA: clinical context–aware
JS^2^E: Jaccard similarity with semantic enrichments
MeSH: Medical Subject Heading
MLP: multilayer perceptron
PICO: Patient/Problem, Intervention, Comparison, and Outcome
PQR: prognosis quality recognition
RNN: recurrent neural network
SVM: support vector machine
