# Supplementary material for: Clinical Context–Aware Biomedical Text Summarization Using Deep Neural Network: Model Development and Validation
Source: J Med Internet Res. 2020 Oct 23;22(10):e19810. doi: 10.2196/19810 (PMC7647812; doi:10.2196/19810)
Supplement: Multimedia Appendix 1 [file jmir_v22i10e19810_app1.docx]

# Multimedia Appendix 1: Survey Questionnaire.

Questionnaire to gauage importance of external resources For Evidence collection of Neurological Diseases

Objective

To determine the ranking and credibility of external research evidence for the prognosis of neurological diseases.

Required Time: **5-7 Minutes,** No. of Questions**: 2**

Brief Description of Study

We are developing research prototype of an automated system to summarize research evidence, collected from credible published sources, to aid clinicians making informed decisions with respect to prognosis of neurological diseases. In order to identify the best resources and to rank them, we would like to collect opinion of physicians who regularly collect evidence from biomedical literature for either education purpose or decision-making process. This study is being submitted in Journal of Medical Internet Research (JMIR). Note: This is purely academic research and authors don’t have any financial motives. Your responses will be only used to give weights to different parameters in proposed algorithms.

**Question 1:** The following table shows the ranking of study types with respect to prognosis in neurology and neuro-surgery domain. Please write your answer to score these study types **(10 highest, 1 Lowest)**.

| **Study Types** | **Score** |
| --- | --- |
| Systematic Reviews of RCTs | 9 |
| Meta-Analysis of RCTs | 8 |
| Randomized Controlled Trials (RCTs) | 10 |
| Meta-analysis of CTs | 5 |
| Systematic Review of CTs | 7 |
| Control Trials (CT) | 6 |
| Cohort Study/ Case-control study/report | 4 |
| Guidelines | 5 |
| Opinion | 2 |
| Observational Study | 3 |
| Any other publication type | 1 |

**QUESTION 2:** THE FOLLOWING TABLE SHOWS THE CREDIBILITY RANKING OF PUBLICATION VENUE IN NEUROLOGY AND NEURO-SURGERY DOMAIN. PLEASE WRITE UP-TO FIVE PUBLISHING VENUE’S NAME (E.G. JOURNAL OF NEUROSURGERY) IN TOP-DOWN ORDER WITH TOP BEING MORE CREDIBLE FOR EVIDENCE EXTRACTION.

| **S. No.** | **Venue (Journal)** |
| --- | --- |
|  | Journal of Neurosurgery |
|  | Neurosurgery |
|  | Annals of Neurology (green Journal) |
|  | Neurology, Neurosurgery, and Psychiatry |
|  | **Stroke** |
